# The association of peritoneal dialysis and hemodialysis on mitral and aortic valve calcification associated mortality: a meta-analysis

**DOI:** 10.1038/s41598-024-55326-9

**Published:** 2024-02-27

**Authors:** Kuan-Jung Chiu, Szu-Chia Chen, Wei-Yu Su, Yong-Yuan Chang, Kai-Chao Chang, Chiu Hui Li, Ying-Jhen Wu, Da-Wei Wu, Chao-Hung Kuo

**Affiliations:** 1 School of Medicine, Kaohsiung Medical University, Kaohsiung, 807 Taiwan; 2 Division of Nephrology, Department of Internal Medicine, Kaohsiung Medical University Hospital, Kaohsiung Medical University, Kaohsiung, 80756 Taiwan; 3 Faculty of Medicine, College of Medicine, Kaohsiung Medical University, Kaohsiung, 807 Taiwan; 4 Department of Internal Medicine, Kaohsiung Municipal Siaogang Hospital, Kaohsiung Medical University Hospital, Kaohsiung Medical University, Kaohsiung, 812 Taiwan; 5 Teaching and Research Center of Kaohsiung Municipal Siaogang Hospital, Kaohsiung, 812 Taiwan; 6 Research Center for Precision Environmental Medicine, Kaohsiung Medical University, Kaohsiung, 807 Taiwan; 7 Department of General Medicine, Kaohsiung Medical University Hospital, Kaohsiung Medical University, Kaohsiung, 807 Taiwan; 8 Department of Healthcare Administration and Medical Informatics, College of Health Sciences, Kaohsiung Medical University, Kaohsiung, 807 Taiwan; 9 Division of Pulmonary and Critical Care Medicine, Department of Internal Medicine, Kaohsiung Medical University Hospital, Kaohsiung Medical University, Kaohsiung, 807 Taiwan; 10 Doctoral Degree Program, Department of International Business, National Kaohsiung University of Science and Technology, Kaohsiung, Taiwan; 11 Health Management and Occupational Safety and Health Center of Kaohsiung Municipal Siaogang Hospital, Kaohsiung, 812 Taiwan; 12 Doctoral Degree Program, Department of Public Health, College of Health Sciences, Kaohsiung Medical University, Kaohsiung, 807 Taiwan; 13 Division of Gastroenterology, Department of Internal Medicine, Kaohsiung Medical University Hospital, Kaohsiung Medical University, Kaohsiung, 807 Taiwan

**Keywords:** Mitral valve calcification, Peritoneal dialysis, Mortality analysis, Chronic kidney disease, Heart failure, Valvular disease, Risk factors

## Abstract

Cardiac valve calcification (CVC), characterized by the accumulation of calcium in the heart valves, is highly prevalent among patients undergoing dialysis. This meta-analysis aimed to provide an updated summary of recent studies on the prognostic value of CVC in patients undergoing dialysis. We conducted a search of PubMed, Embase, and Web of Science to identify observational studies investigating cardiovascular or all-cause mortality associated with CVC in dialysis patients until March 2023. Hazard ratios (HRs) and the corresponding 95% confidence intervals (CIs) were calculated for the meta-analysis, and the strength and significance of the associations between CVC and mortality outcomes in dialysis patients were assessed. From 6218 initially identified studies, we included 10 critical studies with a total of 3376 dialysis patients in a further meta-analysis. Pooled analyses demonstrated a significant association between CVC and an elevated risk of all-cause and cardiovascular mortality in dialysis patients. In our study, we discovered HRs of 1.592 (95% CI 1.410–1.797) for all-cause mortality and 2.444 (95% CI 1.632–3.659) for cardiovascular mortality. Furthermore, subgroup analysis revealed elevated all-cause mortality among patients with mitral valve calcification (HR 1.572; 95% CI 1.200–2.060) compared to those with aortic valve calcification (HR 1.456; 95% CI 1.105–1.917). Similarly, patients undergoing peritoneal dialysis faced a greater risk for all-cause mortality (HR 2.094; 95% CI 1.374–3.191) than those on hemodialysis (HR 1.553; 95% CI 1.369–1.763). This highlights the possibility of CVC being an independent risk factor for dialysis patients, particularly in relation to mitral valve calcification or peritoneal dialysis.

## Introduction

Renal failure is a global health issue that affects a significant number of people worldwide^1^ Cardiovascular disease (CVD) is the leading cause of morbidity and mortality in patients with end-stage kidney disease (ESKD) undergoing dialysis. Although the association between renal function decline and increased risk of CVD has long been acknowledged, the underlying mechanisms remain unclear owing to their multifactorial and intricate nature^2^. Traditional cardiovascular (CV) risk factors, such as advancing age, tobacco use, hypertension, diabetes mellitus, dyslipidemia, and obesity, are widely recognized for their capacity to induce CVD in both the general population and patients with ESKD. Conversely, CKD-associated non-traditional risk factors related to the secondary consequences of renal failure include fluid overload, electrolyte imbalance, anemia, uremia, and systemic inflammation. Dysregulation of calcium and phosphate metabolism plays a prominent role, resulting in abnormal bone turnover and other CV complications, such as vascular and cardiac valve calcification (CVC)^3^.

CVC is highly prevalent among individuals undergoing dialysis, notably affecting the aortic and mitral valves^4^. Therefore, CVC can be categorized based on the specifically affected valvular area: aortic valve calcification (AVC) and mitral valve calcification (MVC). MVC is also known as mitral annulus or mitral valve leaflet calcification. Rather than being a chronic degenerative process characterized by passive calcium deposition, CVC is considered an active process that is intricately linked to various metabolic and inflammatory processes frequently observed in patients with ESKD^5–7^. Increased cardiovascular shear stress has been observed during ultrafiltration in hemodialysis^8^ and following arteriovenous shunt creation, due to hyperdynamic circulation^9^. These two mechanisms represent a significant difference between patients who undergo hemodialysis and those who do not. The sensing of shear stress by ventricularis layer of the aortic valve induces the endothelial-to-mesenchymal transition of valve endothelial cells. Valve endothelial cells that have undergone endothelial-to-mesenchymal may directly transform into activated valve interstitial cells or alternatively influence the surrounding quiescent valve interstitial cells through the paracrine regulation. Activated valve interstitial cells can differentiate into osteogenic valve interstitial cells which is regulated by transcription factors such as Runx2 and Sox9, leading to the aortic valve calcification^10^. Besides, the retention of serum phosphate in advanced CKD patients, reduction of klotho and 1, 25-dihydroxyvitamin D, upregulation of fibroblast growth factor 23 (FGF-23) and iPTH play an important role in vascular and valve calcification^11–13^. Additional mechanisms include systemic and local inflammation with pro-inflammatory cytokines, oxidative stress, uremic toxins, anemia, malnutrition, and cachexia. These factors contribute to the pathogenesis of CVC and its association with ESKD^7^.

Previous research has indicated an association between CVC and all-cause and CV mortality in dialysis patients^14^. However, the mechanisms underlying this association have not yet been thoroughly evaluated. Traditionally, the poor prognosis associated with CVC has been attributed to the presence of coronary artery disease and atherosclerosis, as these conditions share common risk factors. However, a recent study highlighted that CVC is linked to a worsened hemodynamic profile, possibly due to the development of valvular heart disease or diastolic dysfunction^15^. Moreover, several retrospective and prospective cohort studies investigating this relationship have recently been published, the majority of which have demonstrated a correlation between CVC and mortality^16–22^, whereas others have reported conflicting results^23–25^. Therefore, this systematic review and meta-analysis aimed to provide an updated summary of recent studies on the prognostic value of CVC in dialysis patients.

## Methods

### Search strategy

This study was conducted in accordance with the Preferred Reporting Items for Systematic Reviews and Meta-Analyses (PRISMA) guidelines. A systematic search of PubMed, EMBASE, and Web of Science was performed using the following keywords and synonyms: renal dialysis, kidney failure, renal replacement therapy, heart OR cardiac OR valve OR valvular, and calcification OR calcified. The search strategy included Boolean searches and medical subject heading (MeSH) terms. Details of the electronic search strategy are provided in Supplementary Table 1. The last search was performed in March 2023.

### Study selection

Studies that met the following criteria were included: (1) observational cohort studies, including prospective, retrospective, and bidimensional studies; (2) studies limited to human subjects; and (3) those reporting adjusted hazard ratios and corresponding 95% confidence intervals (CIs) of CV or all-cause mortality due to CVC in dialysis patients. Letters, comments, protocols, case reports, case series, reviews, and animal studies were excluded from analysis. Studies with insufficient data were excluded from analysis.

### Data extraction

Two authors independently analyzed each article and performed data extraction. In cases of disagreement, a third investigator was consulted to reach consensus. The following data were extracted from each study: article name, first author, publication year, study design, country of origin, sample size, dialysis vintage and type, sex, age, detection methods and prevalence of CVC, follow-up time endpoint, and covariate adjustments.

### Quality assessment

Quality assessment was performed using the Newcastle–Ottawa Scale (NOS) for observational studies, which comprises three domains (selection, comparability, and outcome) with a maximum score of 9 points. Risk of bias was categorized into three groups: good, fair, and poor. The results were described in Table 1. Two reviewers independently assessed the quality of each study.

**Table 1:** Quality assessment using Newcastle–Ottawa Scale (NOS).

Study, years	Selection				Comparability		Outcome			Total score	Quality
	Representativeness of Exposed cohort	Selection of Non-exposed cohort	Ascertainment of exposure	Outcome not present at start of study	a	b	Assessment of outcome	Follow-up long enough	Adequacy of follow-up		
Cheng et al.^16^	★		★	★	★	★	★	★		7	Good
Bai et al.^17^	★		★		★	★	★	★		6	Fair
Liao et al.^23^	★		★		★	★	★	★		6	Fair
Zhu et al.^19^	★		★	★	★	★		★		6	Fair
Shen et al.^18^	★	★	★		★	★		★	★	7	Good
Chen et al.^24^	★		★		★	★		★		5	Fair
Takahashi et al.^20^	★		★	★	★	★	★	★	★	8	Good
Raggi et al.^25^	★	★	★		★	★	★	★	★	8	Good
Panuccio et al.^21^	★		★		★	★	★	★		6	Fair
Wang et al.^22^	★		★		★	★	★		★	6	Fair

(a) The study controls for age (one star).

(b) Study controls for other factors (one star).

### Statistical analysis

Comprehensive meta-analysis software (version 3.0) was used for statistical analysis. Risk estimates were calculated using adjusted hazard ratios (HRs) and 95% CI. Cochran’s Q test was performed, and Q and *p* values were used to evaluate the heterogeneity among the studies. Statistical significance was set at *p* value < 0.05. Pooled effects were calculated, and a two-sided *p* value < 0.05 was considered significant. Subgroup analysis was also performed to further assess the association between CVC and the risk of mortality according to clinical characteristics. Sensitivity analysis was performed by excluding one study at a time to test the robustness of the pooled results. Publication bias was assessed using the Egger’s test. Statistical significance was set at *p* value < 0.05. In addition, we used a funnel plot test to assess publication bias.

## Results

### Search results

**Figure 1:**
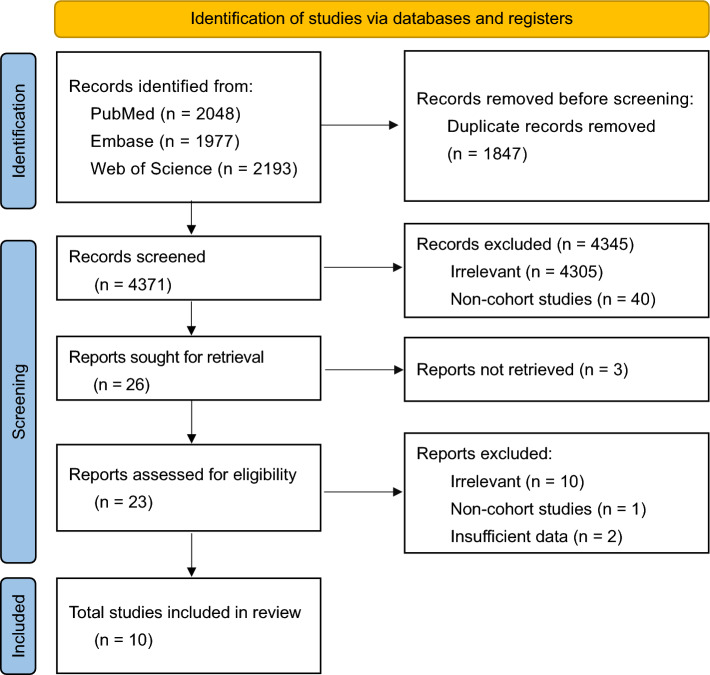
PRISMA (Preferred Reporting Items for Systematic Reviews and Meta-Analyses) 2020 flow diagram of study selection.

In total, 6218 studies were identified in the database search. After the initial screening of titles and abstracts, 23 full-text reports were included and assessed for eligibility. Of these, 10 (3376 patients) were included in the quantitative analysis. The Preferred Reporting Items for Systematic Reviews and Meta-Analyses (PRISMA) flow diagram of the study selection process is shown in Fig. 1. Quality assessment using the NOS for observational studies is described in Table 1. Four studies were classified as good quality, six as fair quality, and none as poor quality.

### Baseline characteristics

Baseline patient characteristics are shown in Table 2 Six studies were prospective cohort studies and four were retrospective cohort studies. Two studies focused on peritoneal dialysis (PD), while others investigated patients undergoing hemodialysis (HD). The percentage of males ranged from 49.3 to 69.9%. Eight studies have investigated Asian patients. The average age of the patients ranged from 55.4 to 68.6 ± 12.6 years. Three studies focused on incident dialysis patients, and the dialysis duration of the other ranged from 2.5 to 8.12 ± 7.62 years. Most studies have defined cardiac calcification as bright echoes of > 1 mm on one or more cusps of the aortic valve, mitral valve, or mitral annulus. The prevalence of CVC ranged from 23.3 to 80.3%, with a mean prevalence of 47.87%. The median follow-up duration ranged from 1.5 to 6.3 years. Three studies only mentioned all-cause mortality as the primary outcome, whereas the other studies reported both all-cause and CV mortalities. Potential confounding factors influencing mortality were adjusted in each study.

**Table 2:** Baseline characteristics of the 10 studies included in the final analysis.

Article	Design	Patient (% male)	Region	Age (years)	Median dialysis time (years)	Detection method	Prevalence of CVC (%)	Median follow-up (years)	Outcome measurement	Adjustment for covariates
Cheng et al.^16^	Prospective cohort	HD, 224 (66.1%)	China	57.4 ± 15.0	Incident	Echocardiography	47.6	4	All-cause death CV death	Age, CVD, Hb, ALB, Ca, P, iPTH
Bai et al.^17^	Retrospective cohort	HD, 434 (62.2%)	China	53.1 ± 13.4	3.43 ± 3.27	Echocardiography	47.2	6.3	All-cause death CV death	Age, DM, CVD, Phosphorus-binding medications, Vitamin D medications, LAD, LVMI, LVEF, Hb, β2-microglobulin, ALB, iPTH
Liao et al.^23^	Retrospective cohort	HD, 297 (52.5%)	Taiwan	68.6 ± 12.6	8.12 ± 7.62	Echocardiography	44.1	3	All-cause death CV death	Age, Sex, CAD, ALB, Transferrin saturation, PTH, Antiplatelet
Chen et al.^24^	Retrospective cohort	PD, 310 (57.7%)	China	57 ± 15.9	3.35 ± 2.125	Echocardiography	23.5	3.4 ± 2.1	All-cause death CV death	Age, P, ALB, eGFR, DM, LVEF
Zhu et al.^19^	Retrospective cohort	HD, 173 (69.9%)	China	58	Incident	Echocardiography	80.3	3	All-cause death	Age, HTN, DM, CVR, LADI, Hb
Chen et al.^24^	Prospective cohort	HD, 110 (58.2%)	China	55.2 ± 1.4	2.5	Echocardiography	25.5	3.5	All-cause death	Age, Sex, ALB, 25(OH)D, AAC
Takahashi et al.^20^	Prospective cohort	HD, 1290 (64.3%)	Japan	61 ± 13	incident	Echocardiography	57.5	4.3	All-cause death CV death	Age, DM, BMI, ALB, creatinine, Ca, LVEF, CRP
Raggi et al.^25^	Prospective cohort	HD, 144 (49.3%)	USA	55.4	2.6	Echocardiography + EBCT	57.6	5.6	All-cause death	Age, Sex, race, DM, atherosclerotic disease, pulse pressure
Panuccio et al.^21^	Prospective cohort	HD, 202 (55.9%)	Italy	59 ± 15	3.58	Echocardiography	23.3	3.7 ± 1.9	All-cause death CV death	Age, Sex, DM, CRP, ADMA, CVD, background CV complication, LVMI
Wang et al.^22^	Prospective cohort	CAPD, 192 (51.0%)	Hong Kong	55 ± 12	3.25 ± 2.58	Echocardiography	32.3	1.5	All-cause death CV death	Age, Sex, dialysis vintage, DM, atherosclerotic vascular disease, hsCRP

*BMI* body mass index, *DM* diabetes mellitus, *CAD* coronary artery disease, *CVD* cardiovascular disease, *HTN* hypertension, *HD* hemodialysis, *Hb* hemoglobin, *LDL-C* low-density lipoprotein cholesterol, *HDL-C* high-density lipoprotein cholesterol, *hsCRP* high sensitivity C-reactive protein, *iPTH* intact parathyroid hormone, *eGFR* estimated glomerular filtration rate, *AKP* alkaline phosphatase, *25(OH)D* 25‐hydroxy Vitamin D, *AAC* Aortic artery calcification, *ALB* albumin, *Ca* calcium, *P* phosphorus, *ADMA* plasma asymmetric dimethyl arginine, *CVR* cardiac valve regurgitation, *MS* mitral valve stenosis, *MR* mitral valve regurgitation, *AR* aortic valve regurgitation, *AS* aortic valve stenosis, *TR* tricuspid valve regurgitation, *LVEF* left ventricular ejection fraction, *LVMI* left ventricular mass index, *LADI* left atrial diameter index.

### Association between cardiac valve calcification and all-cause mortality

**Figure 2:**
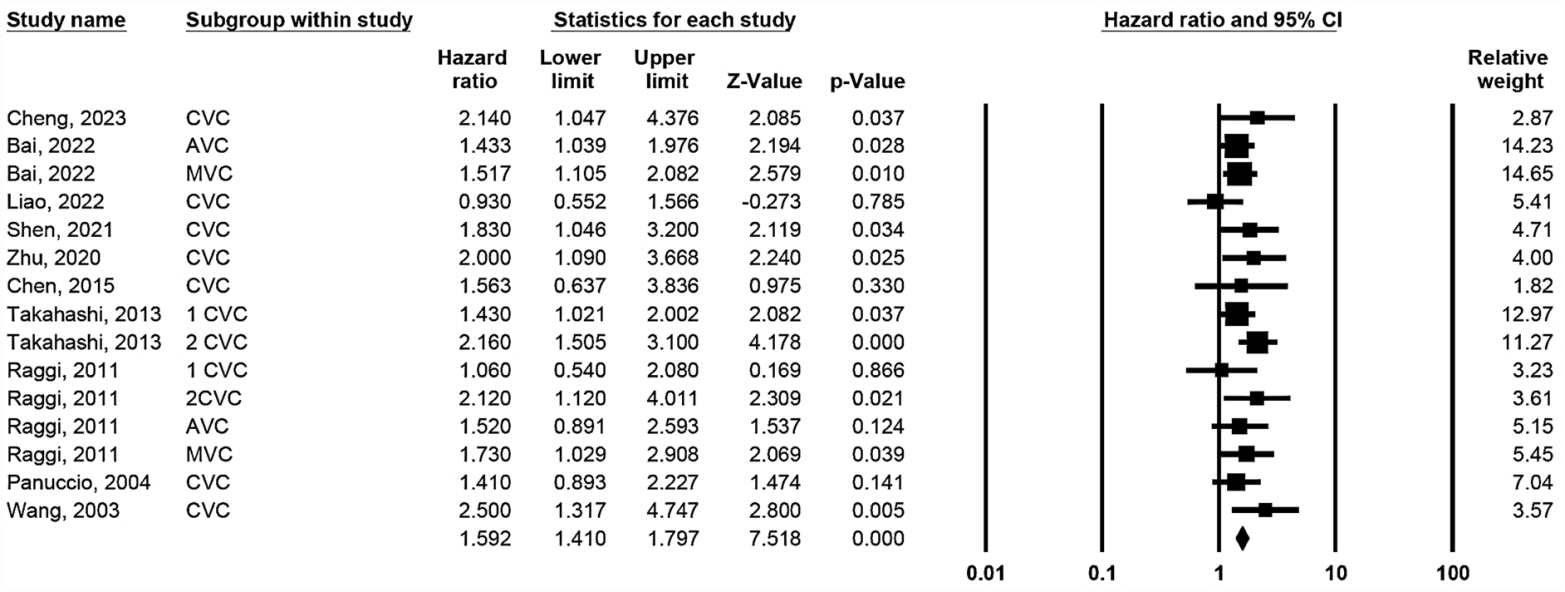
Forest plot of hazard ratios for the association between cardiac valve calcification and all-cause mortality.

Ten studies reported the all-cause mortality rates of MVC or AVC without significant heterogeneity (*p* = 0.478, I-square = 0%). Pooled results in a fixed-effects model showed an association between CVC and mortality (Fig. 2, HR 1.592; 95% CI 1.410–1.797; *p* < 0.001).

### Association between cardiac valve calcification and cardiovascular mortality

**Figure 3:**
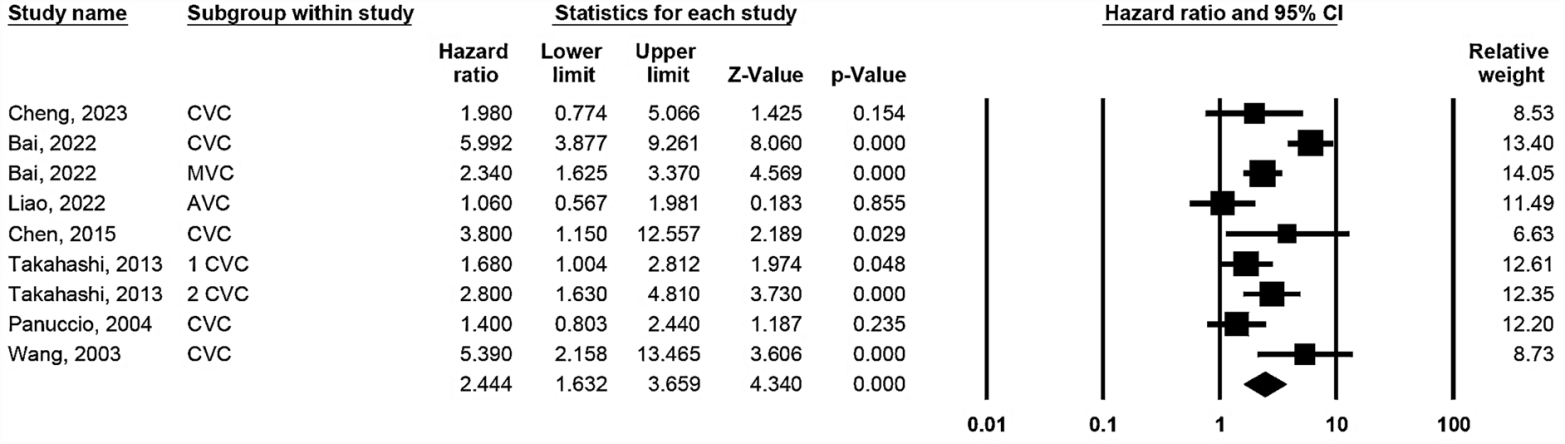
Forest plot of hazard ratios for the association between cardiac valve calcification and cardiovascular mortality.

Seven studies reported CV mortality rates of MVC or AVC with heterogeneity (*p* ≤ 0.001, I-square = 75.68%). Pooled results in a random-effects model showed an association between CVC and CV mortality (Fig. 3, HR 2.444; 95% CI 1.632–3.659; *p* < 0.001).

### Subgroup analyses

Subgroup analysis of the type of CVC demonstrated that MVC (HR 1.572; 95% CI 1.200–2.060; *p* < 0.001) was associated with a greater risk of all-cause mortality than AVC (HR 1.456; 95% CI 1.105–1.917; *p* = 0.008) (Table 3). Subgroup analysis of dialysis type showed that PD (HR 2.094; 95% CI 1.374–3.191; *p* < 0.001) was associated with a greater risk of all-cause mortality than HD (HR 1.553; 95% CI 1.369–1.763; *p* < 0.001) (Table 3). Also, we demonstrated that the number of calcified valves is related to risk of all-cause mortality. Dual valve calcification (HR 2.150; 95% CI 1.570–2.944; *p* < 0.001) showed a higher risk than single valve calcification (HR 1.495; 95% CI 1.299–1.721, *p* < 0.001).

**Table 3:** Subgroup analyses of all-cause mortality.

Subgroup	Studies			HR (95% CI)	*p* value	Heterogenicity (*p* value)
Total		10				
Patients	HD	8	Cheng et al.^16^, Bai et al.^17^, Liao et al.^23^, Zhu (2020), Chen et al.^24^, Takahashi et al.^20^, Raggi et al.^25^, Panuccio et al.^21^	1.553 (1.369–1.763)	< 0.001	0.500
	PD	2	Chen et al.^24^, Wang (2002)	2.094 (1.374–3.191)	< 0.001	0.472
Calcified valve	AVC	2	Bai et al.^17^, Raggi et al.^25^	1.456 (1.105–1.917)	0.008	0.853
	MVC	2	Bai et al.^17^, Raggi et al.^25^	1.752 (1.200–2.060)	< 0.001	0.672

*AVC* aortic valve calcification, *MVC* mitral valve calcification.

### Publication bias

**Figure 4:**
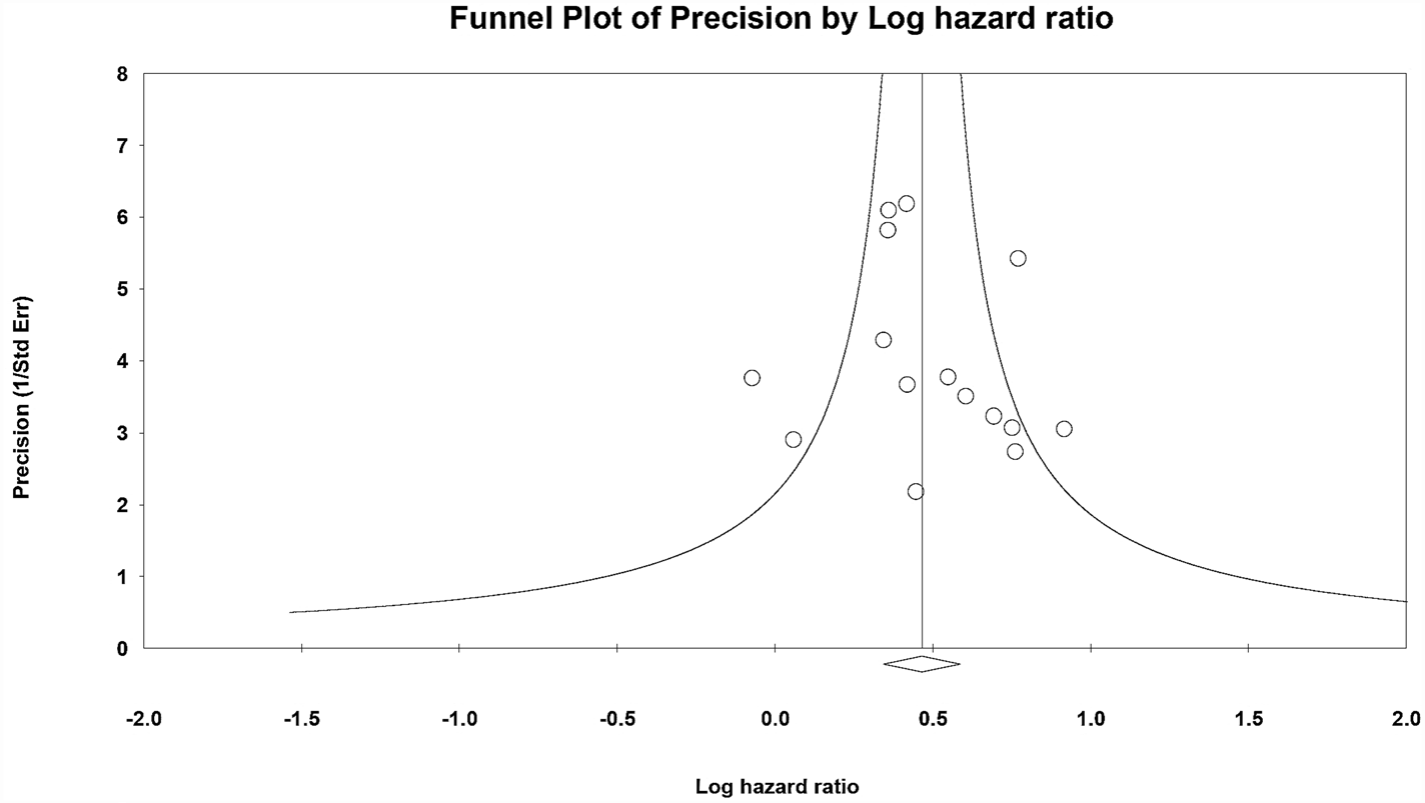
Funnel plot by log hazard ratio for all-cause mortality.

Funnel plots and Egger’s test showed no publication bias in the meta-analysis of all-cause mortality (Fig. 4; Egger’s test, *p* = 0.527). In addition, Egger’s test showed no publication bias in the meta-analysis of CV mortality (*p* = 0.897) or subgroup analysis of CVC and MVC (*p* = 0.315). More information was described in Supplementary Table 2.

### Sensitivity analysis

We performed sensitivity analyses by excluding the articles with the largest weights and repeating the primary analyses. The results were consistent across different analyses, indicating the robustness of the observed outcomes.

## Discussion

This meta-analysis demonstrated the prognostic value of CVC in all-cause mortality and CV mortality in patients undergoing dialysis, particularly in those with MVC or those undergoing PD. Moreover, and increased number of calcified valves is associated with a higher mortality risk. CVC is highly prevalent owing to the dysregulation of bone mineral metabolism in dialysis patients. The clinical presentation of CVC encompasses a wide range of manifestations and often overlaps with symptoms of heart failure and anemia, which are also common in dialysis patients^4,9,26^. Therefore, it is challenging to precisely calculate the prevalence of CVC. The current diagnostic gold standard for CVC is transthoracic echocardiography, which is consistent with the findings of most studies included in the analysis. The reported prevalence of valvular calcification ranges from 23 to 59%, with an increasing prevalence as renal function declines^7,14^. The prevalence in the included studies ranged from 23.5 to 80.3%. Although the upper limit is higher than that reported in previous studies, if we exclude this outlier, the prevalence ranges from 23.5 to 57.6%, which is consistent with the results of previous studies. Severe CVC often progresses to valvular heart disease and worsens hemodynamic profile. However, the prognostic value of CVC remains controversial. Our primary outcomes showed that CVC was associated with higher rates of all-cause mortality and CV mortality among patients undergoing dialysis, with HRs of 1.592 (95% CI 1.410–1.797) and 2.444 (95% CI 1.632–3.659), respectively. Besides, dual CVC presents a higher risk compared to single CVC. These findings are consistent with those of a previous study by Wang et al.^14^, which included relatively recently published retrospective and prospective studies. The HRs for all-cause and CV mortality were slightly lower than those reported in previous studies (1.592 and 1.73, respectively, and 2.444 and 2.81, respectively). Cochrane’s Q test showed higher heterogeneity in the results of CV mortality, which may be due to the limited number of studies included in this analysis.

Subgroup analyses revealed that patients with MVC had a higher risk than those with AVC did. This finding was not reported in a previous meta-analysis of dialysis patients^14^. Renal failure is associated with left-sided valvular disease, especially aortic stenosis and mitral regurgitation, resulting in a poor prognosis. Therefore, we focused on the association between progressive calcification and left valvular disease. Several meta-analyses have indicated an association between AVC and MVC and worse outcomes in the general population with or without CVD^27–30^. While AVC and MVC share similar contributing factors, such as aging, abnormal calcium and phosphate metabolism, and systemic atherosclerosis^31^, previous studies have reported differences in the underlying mechanisms. Hensen et al. reported that although AVC was more prevalent than MVC in both dialysis patients and the general population, MVC was independently associated with more advanced CKD stages and higher all-cause mortality, whereas AVC did not show such associations. The authors suggested that this difference may be due to delayed diagnosis and lower rates of valve intervention therapy owing to the subtle symptoms of MVC^32^. Furthermore, an association has been observed between MVC and elevated levels of serum β2-microglobulin (β2M)^33^, a substance mainly eliminated by the kidneys that tends to accumulate in the myocardium, aortic and mitral valves, and other cardiac tissues in patients undergoing HD^34^. In addition to being an indicator of the clearance of middle molecules by HD, serum β2M is considered a marker of inflammation and is related to an increased risk of all-cause mortality, long-term HD, dyslipidemia, and malnutrition, all of which have unfavorable effects on CV outcomes^33^. In addition, studies of patients undergoing PD have implied a similar result of higher C-reactive protein levels in MVC-positive patients^35^, and that diabetes mellitus can serve as a risk factor for MVC^36^. Another risk factor for MVC is increased mitral stress, which accelerates the calcification process and is associated with hypertension, aortic stenosis, hypertrophic cardiomyopathy, and other diseases that increase the left ventricular systolic pressure. Finally, MVC is associated with valvular diseases and other CV complications including atrial fibrillation^37,38^, arrhythmia due to mitral annulus conduction abnormalities^39^, and endocarditis^40^. In summary, aspects related to the diagnosis, pathophysiology, and occurrence of CV complications may contribute to the association between AVC and MVC incidence and mortality. A recent meta-analysis evaluated the correlation between CVC and prognosis of patients with CKD. The results showed that both MVC and AVC had predictive value and that MVC had a higher risk, which is also consistent with our findings^41^. Further evaluation is necessary to determine the underlying mechanisms.

The prevalence of CVC varies among patients undergoing different dialysis modalities, ranging from 19 to 84% in those undergoing HD patients to 32–47% in those undergoing PD^42^. Our results showed that the average prevalence of CVC was lower in PD patients than in HD patients (26.9% and 51.5%, respectively), this finding corresponds with another study^43^. However, in the subgroup analysis, the HR is higher in PD patients than in HD patients. A previous meta-analysis showed the same trend^14^, and a cohort study reported that PD was associated with a higher risk of myocardial infarction and CV mortality than HD^44^ and that this effect persisted beyond the first year of treatment^45^. However, another study found no significant differences in the risk of mortality, de novo CV disease, or ischemic heart disease between patients undergoing HD and PD. Nevertheless, HD is associated with a high risk of congestive heart failure^46,47^. Recently, a meta-analysis demonstrated comparable mortality rates and adverse CV events between PD and HD^48^. Conversely, another recent meta-analysis found that PD patients had lower rates of CV events, such as congestive heart failure, atrial fibrillation, and peripheral arterial disease, but higher mortality rates than HD patients^49^. Several hypotheses have been proposed to explain the conflicting results. Infusion of dialysis solution into the peritoneal cavity increases intraperitoneal pressure and systolic blood pressure, which may be associated with fluid overload and hemodynamic changes that increase CV risk^50^. On the other hand, the rapid removal and exchange of body fluid during HD contribute to hemodynamic instability^51^, and the arteriovenous fistula used in HD patients increases cardiac preload and leads to congestive heart failure^49^. Therefore, the variable mortality between HD and PD may related to patient-specific factors and selection bias. Although CVC is a marker of higher mortality and cardiovascular mortality in this population, the effect is not independent of other traditional CV risk factors^21^, such as age, diabetes, hypercalcemia and hyperphosphatemia^52,53^. Previous study also suggest higher glucose levels in PD patients can lead to insulin resistance, and also alterations in glycation products may increase CV damage^54^. Inconsistent definitions of cardiac mortality may also have influenced the statistical results of different studies^55^. In addition, the small number of patients from only two studies included in our analysis is a limitation. In summary, the association between dialysis modality and CV mortality remains controversial and is affected by CVC and other factors^56^. Our results showed CVC is less frequent in peritoneal dialysis than in HD, but its presence still indicates a greater prognostic value. Additional studies are required to confirm the association between CVC and dialysis modality.

Owing to the potential risks to the CV system, the CVC should be appropriately controlled in dialysis patients. However, methods for reversing the impact of calcified valves are limited. The aim of the current medical treatment of CVC emphasizes the control and prevention of CKD-MBD, namely, the stabilization of the serum levels of calcium, phosphate, and PTH^42,57^. Calcimimetics, calcitriol, and vitamin D analogs are used to treat secondary hyperparathyroidism in dialysis patients^58^. Although vitamin D analogs decrease serum PTH levels, they also induce calcification by elevating serum calcium and phosphate levels^59^. Therefore, calcimimetics such as cinacalcet activate receptors on the parathyroid gland and thus lower the levels of PTH, serum calcium, and phosphate have been introduced. However, a randomized controlled trial and meta-analysis did not find significant reductions in all-cause mortality, CV mortality, or major adverse CV events in dialysis patients receiving cinacalcet therapy^60,61^. Calcium-based and non-calcium-based phosphate binders are widely used^62^. Non-calcium-based phosphate binders, such as sevelamer, were found to be associated with lower all-cause mortality than calcium-based phosphate binders in one study^63^, whereas another study found no significant benefit on CV mortality^64^. Owing to the failure of traditional therapies, novel treatments have emerged to control CVC in patients with CKD or on dialysis. For example, serum alkaline phosphate levels have been correlated with CVC in HD patients^65^, and serum albumin levels have been shown to be predictors of CVC severity in dialysis patients^66^. In addition, a novel calcification inhibitor, SNF472, has entered clinical trials^67^.

This study had several limitations. First, because of the relatively strict inclusion criteria and the limited number of previous studies, the analysis included only 10 studies, and the majority of patients included in the studies were from Asia, which may have led to publication bias and restricted the applicability of this meta-analysis to different populations. Second, the adjustments for covariates between the studies were different, and the heterogeneity in the comparisons of CV mortality may have influenced the robustness of the results. Third, the relatively short median dialysis time may have prevented the detection of certain CV diseases, which typically require a long period of development.

## Conclusion

This article summarizes recent findings regarding the association between CVC and prognosis in dialysis patients and reviews the factors and mechanisms involved in the development of CVC. These results indicate that CVC is associated with higher all-cause and CV mortality in patients undergoing dialysis, whether HD or PD patients. Patients with MVC had a higher all-cause mortality than those with AVC. Further research is required to develop new treatments and to elucidate the mechanisms involved in the development of CVC.

### Supplementary Information

Supplementary Tables.

## Data Availability

All data generated or analyzed during the present study are included in this published article.
