# Supplementary material for: The association of peritoneal dialysis and hemodialysis on mitral and aortic valve calcification associated mortality: a meta-analysis
Source: Sci Rep. 2024 Feb 27;14:4748. doi: 10.1038/s41598-024-55326-9 (PMC10899208; doi:10.1038/s41598-024-55326-9)
Supplement: Supplementary file 1 — Supplementary Tables. [file 41598_2024_55326_MOESM1_ESM.docx]

**Supplementary Table** **1.** **Electronic search strategies**

| **PubMed: N=2048** | | |
| --- | --- | --- |
| Population | #1 | ("Renal Dialysis"[Mesh] OR "Kidney Failure, Chronic"[Mesh] OR dialysis OR end stage renal disease) |
| Intervention | #2 | ("Heart"[Mesh] OR heart OR cardiac OR valvular OR valve OR mitral OR aortic) |
| Intervention | #3 | (calcification OR calcified) |
| Search algorithm | #4 | #1 AND #2 AND 3# |
| **Embase: N=1977** | | |
| Population | #1 | ('renal replacement therapy'/exp OR 'renal replacement therapy' OR 'end stage renal disease'/exp OR 'end stage renal disease') |
| Intervention | #2 | ('heart disease'/exp OR 'heart disease' OR 'valvular heart disease'/exp OR 'valvular heart disease') |
| Intervention | #3 | ('calcification'/exp OR calcification) |
| Search algorithm | #4 | 1# AND 2# AND 3# |
| **Web of Science: N=2193** | | |
| Population | #1 | dialysis OR end stage renal disease |
| Intervention | #2 | heart OR cardiac OR valvular OR valve OR mitral OR aortic |
| Filters | #3 | calcification OR calcified |
| Search algorithm | #4 | 1# AND 2# AND 3# |

**Supplementary Table 2. Egger’s regression intercept**

| **All-cause mortality** | | **Cardiovascular mortality** | | **Subgroup analysis** | |
| --- | --- | --- | --- | --- | --- |
| Intercept | 0.56352 | Intercept | -029634 | Intercept | 0.86937 |
| Standard error | 0.86765 | Standard error | 2.21426 | Standard error | 0.65313 |
| 95% lower limit (2-tailed) | -1.31092 | 95% lower limit (2-tailed) | -5.53213 | 95% lower limit (2-tailed) | -1.94082 |
| 95% upper limit (2-tailed) | 2.43795 | 95% upper limit (2-tailed) | 4.93966 | 95% upper limit (2-tailed) | 3.67955 |
| t-value | 0.64948 | t-value | 0.13379 | t-value | 1.33108 |
| df | 13 | df | 7 | df | 2 |
| P-value (2-tailed) | 0.52734 | P-value (2-tailed) | 0.89734 | P-value (2-tailed) | 0.31462 |
